# Preliminary Investigation of the Effect of Maceration Procedures on Bone Metabolome and Lipidome

**DOI:** 10.3390/metabo12111020

**Published:** 2022-10-25

**Authors:** Andrea Bonicelli, William Cheung, Sheree Hughes, Daniel J. Wescott, Noemi Procopio

**Affiliations:** 1 The Forensic Science Unit, Faculty of Health and Life Sciences, Ellison Building, Northumbria University, Newcastle upon Tyne NE1 8ST, UK; 2 Department of Applied Sciences, Faculty of Health and Life Sciences, Northumbria University, Newcastle upon Tyne NE1 8ST, UK; 3 Department of Forensic Science, College of Criminal Justice, Sam Houston State University, Huntsville, TX 773402525, USA; 4 Forensic Anthropology Center at Texas State, Department of Anthropology, Texas State University, San Marcos, TX 78666, USA

**Keywords:** maceration, metabolomics, lipidomics, forensic, bone

## Abstract

The study of post-mortem changes is a crucial component of forensic investigation. Human forensic taphonomic facilities (HFTFs) are the only institutions allowing the design and execution of controlled human decomposition experiments. When bodies are skeletonized, bones are normally stored in skeletal collections and used for anthropological studies. However, HFTFs apply chemical and/or thermal treatments to the remains prior bone long-term storage. These treatments are believed to alter heavily the original biochemical and molecular signature of bone material. The present study aims to evaluate the effect of these procedures on the bone metabolome and lipidome by using an animal bone model. Three intact bovine tibiae were processed using three protocols routinely applied at HFTFs, and their three counterparts were used as non-treated controls. Bone powder samples were subjected to biphasic extraction and both metabolites and lipids were analysed via liquid chromatography tandem mass-spectrometry. Results showed severe reductions in the abundances of both metabolites and lipids, and the presence of contamination introduced by cleaning agents. Despite the preliminary nature of the study, we demonstrated that the biochemical profile of bone is heavily affected by the maceration procedures. Ideally, these treatments should be avoided, or replaced by minimally invasive procedures agreed across HFTFs.

## 1. Introduction

Human forensic taphonomic facilities (HFTFs) are becoming increasingly popular as they allow the comprehensive study of the complex interplay of the intrinsic and extrinsic factors that contribute to cadaveric decomposition. To date, there are eleven HFTFs worldwide including eight in the USA (8), Australia (1), Canada (1) and Europe (1), each operating differently depending on the national or state laws regarding treatment and disposition of human remains. The human skeletal remains associated with HFTFs can provide a valuable resource for conducting biomolecular studies that have significant potential for forensic applications such as PMI estimation [1,2,3,4,5,6]. Amongst the available biomolecular fields of investigations for PMI estimation, metabolomics is acquiring a particular attention in the forensic arena. A recent study from Pesko et al. [7] recently applied metabolomics on muscle tissue from human individuals decomposed at the Forensic Anthropology Center at Texas State (FACTS). Results showed that over a PMI of maximum 19 days, xanthine, uracil, choline phosphate, n-acetylneuraminate, and 1-methylnicotinamide could be good metabolomic markers for PMI estimation. Similarly, Wood and Shirley [8] analyzed anterior quadriceps muscle tissue from one donor decomposing at the Forensic Anthropology Center (FAC) at the University of Tennessee and showed that, across 24 days PMI, sterol sulfates, cholines, ethanolamines and phosphatidylglycerols decrease significantly. In contrast, free fatty acids were shown to have a positive relationship with PMI [8]. At the same institution (University of Tennessee Anthropology Research Facility), a larger test involving the lipidomic analysis of vastus lateralis muscle from a total of 31 individuals over 2000 accumulated degree days showed a robust correlation with PMI based on regression, especially for phosphatidylglycerol (PG) 34:0 and phosphatidylethanolamine (PtdE) 36:4 [9]. An unpublished work from Dudzik et al. [10] also proved the applicability of molecular profiling of bone tissue based on lipidomics with mass spectrometry to estimate PMI. They showed that three months are a crucial interval in the decomposition of phosphosphatidylcholine, that could be therefore a useful marker for short PMI estimation.

All the above-mentioned studies clearly show the potential of metabolomics in forensic science, as well as and the importance of HFTFs in providing unvaluable human models for the study of decomposition. However, while there are some commonalities amongst all the facilities, many of the procedures such as chemical and thermal processing skeletal remains to remove soft tissues and fluids are unique to each facility. The lack of common guidelines between HFTFs on the treatment of bones for long-term storage (a procedure known as “soft maceration”) significantly impacts the possibility of conducting biomolecular research on the donated osteological collections across different HFTFs, thereby drastically limiting the potential of obtaining comparable forensic and taphonomic information from the bones when conducting novel research involving state-of-the-art biochemical and molecular methods. As an example, the National Institute of Standards and Technology (NIST) guidelines clearly advise against the use of excessive soaking and the use of heat and chemical agents on human bones [11] as this can damage or destroy bone, teeth, and DNA. It is not known how soft maceration procedures can affect the survival of bone biomolecules (including DNA, proteins, lipids, and metabolites), and therefore the reliability of the results obtained from processed osteological records must be questioned. Furthermore, inconsistencies on the chemical and physical treatment of the bones could make comparison of data between collections nearly impossible and the use of data in casework unreliable. Several studies have so far demonstrated the importance of developing standardized protocols for the extraction of low molecular weight compounds from different tissues and fluids by controlling extraction buffer, temperature and other factors that can affect the stability of metabolites and lipids [12,13,14,15,16]. More specifically, it has been demonstrated that thermal treatment is one of the main factors that can induce the degradation of both compounds [17,18,19]. Due to the increasing interest in the application of small compound profiling for forensic applications, it is essential to evaluate potential sources of biases induced by sample treatment and handling. Moreover, it is fundamental to understand if such metabolomics and lipidomics profiling studies can provide meaningful information when testing macerated bones, to save resources whilst protecting precious human material.

These facilities have now been in use from several decades and keep offering invaluable samples for anthropological research. However, there is a lack of standardized guidelines for the treatment of human remains. For the first time, to the best of our knowledge, we are highlighting an issue that could compromise all future metabolomics studies based on such material. The current study aims to investigate the effect of three different maceration procedures developed on routinely performed protocols at Forensic Anthropology Center at Texas State University (FACTS) facility (n = 1), employing a bath of deionized water in laundry detergent at 87 °C, and from the Southeast Texas Applied Forensic Science (STAFS) facility (n = 2), consisting of a milder bath of detergent and deionized water at 55 °C, on metabolites and lipids extracted from bovine bone tissue and analyzed by LC-MS/MS. Results of the present study have the objective of clarifying whether these protocols are suitable or not to preserve the biomolecular integrity of the bone matrix. In particular, we aim at providing a preliminary understanding on how different protocols could differentially affect the bone metabolomic and lipidomic profile in order to ensure the comparability of studies conducted on material sourced at different HFTFs, to increase the awareness on this issue and to eventually stimulate debates on future change in policies for more appropriate and standardized treatment of human remains.

## 2. Materials and Methods

**Figure 1:**
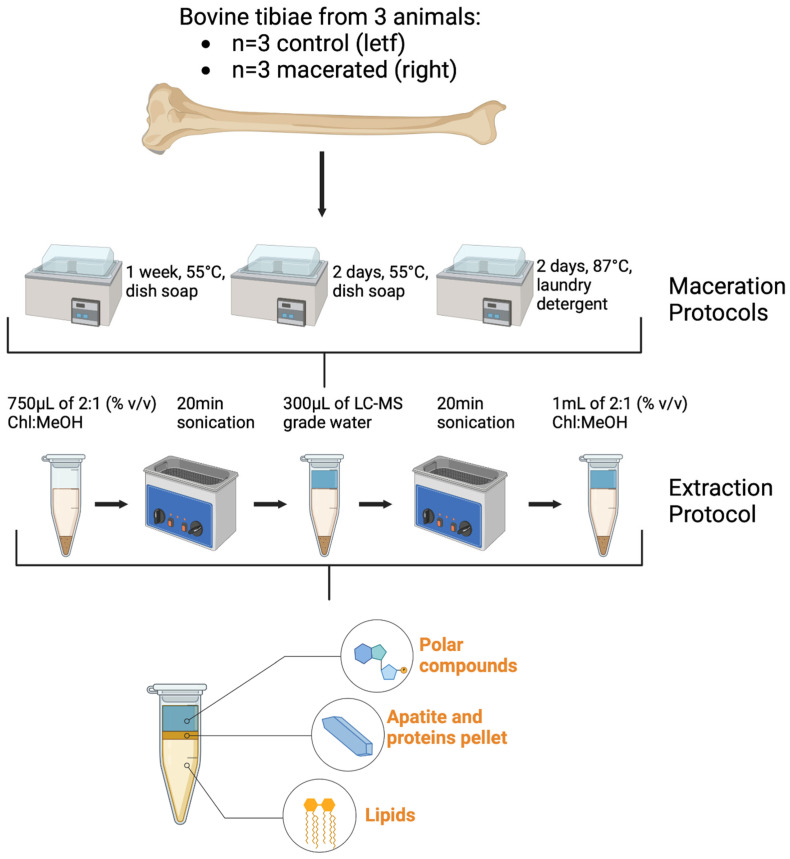
Schematic representation of maceration protocols and compound extraction.

Three pairs of bovine tibiae were purchased from a local butcher and used in the present study. These bovid samples are animal by-products under the guidelines and licensing of the Department for Environment, Food and Rural Affairs (DEFRA). Experimental procedures were conducted within the premise of the DEFRA license. The left bones were sampled in their native state (i.e., non-macerated, fresh) while each of the right bones were subjected to a different maceration protocol: (1) a solution of Dish soap liquid wash:deionized water (1:2% v/v) for one week at 55 °C; (2) Dish soap liquid wash:deionized water (1:2% v/v) for two days at 55 °C; (3) Laundry detergent:deionized water (2 oz/15 L) for two days at 87 °C (Table 1 and Figure 1). The three protocols were adapted from the ones routinely used at FACTS and at STAFS. Powder from the mid shaft posterior cortical surface was obtained by means of a Dremel electric drill equipped with a diamond impregnated bit operated at 10,000 RPM.

### 2.1. Biphasic Extraction

Chloroform (Chl), AnalaR NORMAPUR^®®^ ACS was purchased from VWR Chemicals (Lutterworth, UK). Water Optima™ LC/MS Grade, Methanol (MeOH) Optima™ LC/MS Grade, Pierce™ Acetonitrile (ACN), LC-MS Grade and Isopropanol (IPA), Optima™ LC/MS Grade were purchased from Thermo Scientific (Hemel Hempstead, UK). In total two replicates for each of the six specimens were collected and extracted according to a modified Folch et al. [20] protocol as follows: 25 mg of bone powder was placed in tube A and 750 μL of 2:1 (% v/v) Chl:MeOH were added. Samples were vortexed for 30 s and sonicated in ice for additional 20 min. To induce phase separation, 300 μL of LC-MS grade water was added and sonicated in ice for additional 15 min. The samples were then centrifuged at 10 °C for 5 min at 2000 RPM. The respective upper and lower fractions were collected and transferred to fresh Eppendorf tubes and the samples were re-extracted for a second time using 1 mL of 2:1 (% v/v) Chl:MeOH. The two respective fractions were combined and concentrated. The organic lipid fraction was preconcentrated using a vacuum concentrator at 35 °C for until all the organic solvents was removed. The aqueous metabolite fractions were flash frozen in liquid nitrogen and preconcentrated using a lyophilizer cold trap operating at −65 °C until dry. The respective dry fractions were then stored at −80 °C until further analysis. The metabolite fraction was resuspended in 100 μL in 95:5 ACN/water (% v/v) and sonicated for 15 min and centrifuged for 15 min at 15 K RPM at 4 °C and supernatant was then transferred to 1.5 mL autosampler vials with 200 μL microinsert and caped. 20 μL of each sample were collected and pooled to create the pooled QC. The lipid extracts were resuspended in 100 μL of 1:1:2 (% v/v) water:ACN:IPA, sonicated for sonicated for 15 min and centrifuged for 15 min at 15,000 RPM at 10 °C. The supernatant was then transferred into a 1.5 mL autosampler vials with 200 μL microinsert and caped. 20 μL of each sample were collected and pooled to create the pooled QC. The sample set was then submitted for analysis.

### 2.2. LC-MS/MS Analysis—Metabolomics

The metabolomic characterization of the bone samples was performed on a Thermo Scientific (Hemel Hempstead, UK) Vanquish Liquid chromatography system connected to an ID-X High Resolution Mass Spectrometer (MS) system. The MS data were acquired using the AcquireX acquisition workflow (data dependent analysis). The MS parameters were set as follows: MS1 mass resolution 60 K, for MS2 30 K stepped energy (HCD) 20, 25, 50 scan range 100–1000, RF len (%) 35, AGC gain, intensity was set to 25% and minimum intensity threshold was set to 2^4^ with an injection time of 50 ms. An extraction blank was used to create background exclusion list and a pooled quality control (QC) were used to create the inclusion list. Hydrophilic Liquid Interaction Chromatography (HILIC) separation was performed on a Waters Acquity UPLC BEH amide column (2.1 × 150 mm with particle size of 1.7 μm) operating at 45 °C with a flow rate of 200 μL/min. The liquid chromatography (LC) gradient consisted of a binary buffer system, buffer A (LC/MS grade water) and buffer B (LC/MS grade ACN), both containing 10 mM ammonium formate. Independent buffers systems were used for positive and negative ionization mode acquisition, respectively; for positive ionization mode buffers pH was adjusted using 0.1% formic acid, whereas for negative ionization mode buffers pH was adjusted using 0.1% v/v ammonia solution. The LC gradient was the same for both polarities, namely 95% B at T0 hold for 1.5 min followed by a linear decrease to 50% B in 11 min, hold for 4 min, return to the starting conditions, and hold for further 4.5 min (column stabilization). The voltage applied for positive and negative ionization modes was 3.5 kV and 2.5 kV, respectively. Injection volumes used were 5 μL for positive ionization mode (ESI+) and 10 μL for negative ionization mode (ESI−).

### 2.3. LC-MS/MS Analysis—Lipidomics

The MS data were acquired using the AcquieX acquisition workflow (data dependent analysis). The MS operating parameters were as follows: MS1 mass resolution 60 K, MS2 30 K scan range 250–1250, stepped energy (HCD) 20, 25, 50. RF len (%) 35, AGC gain, intensity threshold 2^4^ 25% custom injection mode with an injection time of 50 ms. The chromatographic separation was performed on a Waters Acquity UPLC CSH C18 column (2.1 × 150 mm with particle size of 1.7 μm part no. 186005298), operating at 55 °C with flow rate of 200 μL/min. The LC gradient consists of a binary buffer system, buffer A (LC/MS grade water/ACN 40/60% v/v) and buffer B (90:10% v/v IPA and ACN) both containing 10 mM ammonium formate. Independent buffers systems were used for positive and negative ionization acquisition modes; for ESI+ buffers pH was adjusted using 0.1% formic acid, whereas for ESI− buffers pH was adjusted using 0.1% ammonia solution. The LC gradients were the same for both polarities: 60% B at T0 hold for 1.5 min followed by a linear increase to 85% B at 7 min, a further increase to 95% B at 12.5 min and hold for 4.5 min, return to starting condition and hold for further 4.5 min (column stabilization). Injection volumes used were 3 μL ESI+ and 5 μL for ESI-. The voltage applied for ESI+ and ESI- was 3.5 kV and 2.5 kV, respectively. The heated Electrospray Ionization (ESI) conditions for 200 μL were as follows: Sheath Gas: 35, Aux Gas 7 and Sweep Gas of 0. Ion Transfer tube Temp was: 300 °C and Vaporizer Temp was 275 °C. The ESI Conditions were the same for both metabolomics and lipidomics.

### 2.4. Data Processing and Statistical Analysis

The metabolomic ESI+ and ESI− data sets were processed via Compound Discoverer 3.2 using the following settings: untargeted metabolomic workflow, parent ion mass tolerance 10 ppm, alignment model adaptive curve, minimum intensity 16 signal to noise ratio (S/N) threshold 0.3 min, compound consolidation, retention time tolerance 0.3 min. Database matching were performed at MS2 level using Thermo Scientific online mzCloud databases (Hemel Hempstead, UK) with a similarity index of 70% or higher.

The lipidomic ESI+ and ESI− data sets were processed via Thermo Scientific Lipid search version 4 using the following settings: high energy collision database (HCD) retention time 0.1 min, parent ion mass tolerance (5 ppm), product ion 10 ppm. Alignment method (max), top rank off, minimum m-score 5.0, all isomer peaks, ID quality filter A and B only. Lipid IDs were matched using LipidSearch (Thermo Scientific, Hemel Hempstead, UK) using in silico library MS2 level. Corresponding metabolomics and lipidomics pooled QCs samples were used to evaluate and exclude instrumental drifts. Any metabolite/lipid features which has a residual standard deviation (RSD) <25% within the QCs were retained and extrapolated to the data set.

### 2.5. Statistical Analysis

Metabolomics and lipidomics data were normalized by mean values and cube root transformed before applying Pareto scaling. Unsupervised principal component analysis (PCA) was employed to evaluate the differences between fresh versus macerated bones, as well as and potential differences between the three maceration protocols. Hierarchical clustering and heatmaps were then employed to obtain an overview of the different compounds across the groups. *T*-test was then used to statistically compare compounds in fresh versus macerated bones due the greater sample size in these two groups. Statistical analysis was carried out in R version 4.2.0 (2022-04-22) with the “MixOmics” package [21].

## 3. Results

**Figure 2:**
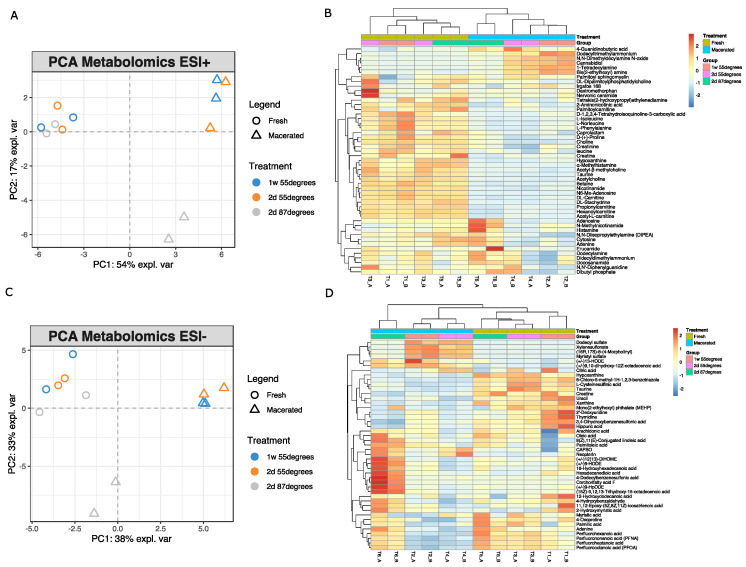
PCA results from metabolites profiled in ESI+ (**A**) and ESI− (**C**) show very similar patterns with clear separations between fresh and macerated bones as well as between protocols involving different temperatures and cleaning agents. Hierarchical clustering (**B**,**D**) confirms the class separation observed with PCAs.

PCA for metabolomics assays, both in positive (ESI+) and negative (ESI−) ionization modes, explained 71% of the total variance. No clear inter-individual differences can be observed between the three individuals, as the non-macerated bones form a unique cluster. However, it is possible to see a clear planar separation on the first dimension between fresh and macerated samples (Figure 2).

Protocols #1 and #2 (differing uniquely by the duration of the procedure) cluster together in the upper right region of the plot, whereas samples processed with protocol #3, which involves a higher water temperature (87 °C), seem to behave differently and clusters in a different quadrant in comparison with the other two groups, suggesting that the second component may retain variation between the different protocols. The hierarchical clustering shows that similar metabolomic profiles are shared by samples subjected to the same maceration treatments. Samples cluster according to their condition (e.g., macerated vs. non-macerated) but also to the specific protocol used, suggesting that all variables (i.e., duration and temperature of the protocol and cleaning agent) play a role in the final metabolomic profile. As can be seen in the heatmap of metabolites analyzed in ESI+ (Figure 2B), the bulk of the metabolites profiled are more abundant in the fresh samples than in the macerated ones. The heatmap also suggests the presence of a cluster of compounds on the top right of the plot that are more abundant in the samples treated with protocols #1 and #2 (55 °C), as well as of another cluster of compounds at the bottom of the plot found more abundant in the bones processed specifically with protocol #3 (87 °C). Very similar same trends can also be identified in ESI−, where clear clusters can be observed for the two main groups (e.g., macerated vs. non-macerated) as well as for the three different maceration protocols (Figure 2D). Several identified molecules are more abundant in the non-treated samples, despite a group of compounds being more abundant in samples treated with protocols #1 and #2 and another group abundant in those treated with protocol #3.

**Figure 3:**
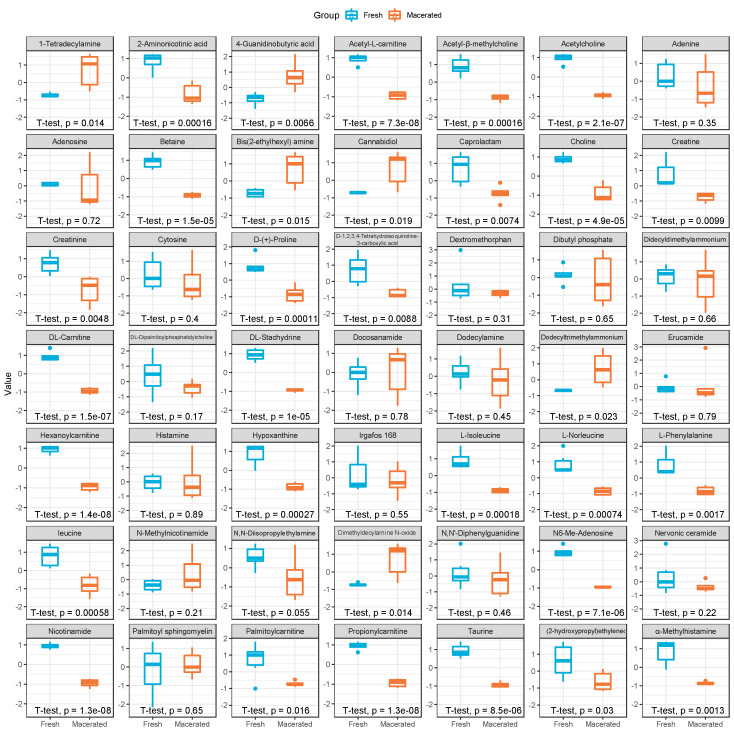
Boxplots comparing fresh (**blue**) and macerated (**orange**) samples profiled in ESI+. Metabolite profiling shows that endogenous compounds are significantly more abundant (paired *t*-test) in the fresh samples, while compounds less abundant are mainly chemical agent present in the cleaning products used during the maceration process.

Compounds identified and selected with ESI+ after the filtration steps reported in the Methods were then evaluated in detail for their abundance in non-macerated vs. macerated samples and their significance level (Figure 3 and Table S1). As previously mentioned, most of the compounds are confirmed to be more highly expressed in the fresh specimens. However, seven compounds are found to be more abundant in the macerated group: 4-guanidinobutyric acid, a degradation product of arginine, Bis (2-ethylhexyl) amine, cannabidiol and dimethyldecylamine N-oxide, not commonly identified as part of endogenous metabolism. N,N-dimethyldecylamine, N-oxide and 1-tetradecylamine, both known to be present in washing products and surfactants, and dodecyltrimethylammonium, another surfactant commonly used as oil-dissolving agent. In ESI− (Figure S1), eight compounds like these are found to be significantly more abundant in the macerated group compared to the fresh counterpart. They include (+/−)9,10-dihydroxy-12-octadecenoic acid, a derivative of linoleic acid diol, that has been reported to be toxic in human’s tissue preparations, myristyl sulfate, an exogenous compound used in cosmetics, dodecyl sulfate, an anionic organic compound used as surfactant, (16R,17S)-8-(4-morpholinyl) and xylene sulfonate, respectively an anionic surfactant and a coupling agent in commercial dishwashing products, 13-hydroxyoctadecadienoic acid (13-HODE), a linoleic acid oxidation product that has vasoactive properties, neopterin, a catabolic product of guanosine triphosphate, and 3-(cyclohexylamino)-2-hydroxy-1-propanesulfonic acid (CAPSO), a chemical used as buffering agent in biochemistry. In contrast, the metabolites more abundant in the fresh samples, in both ionisation modes, are mostly endogenous compounds normally involved in tissue metabolism.

**Figure 4:**
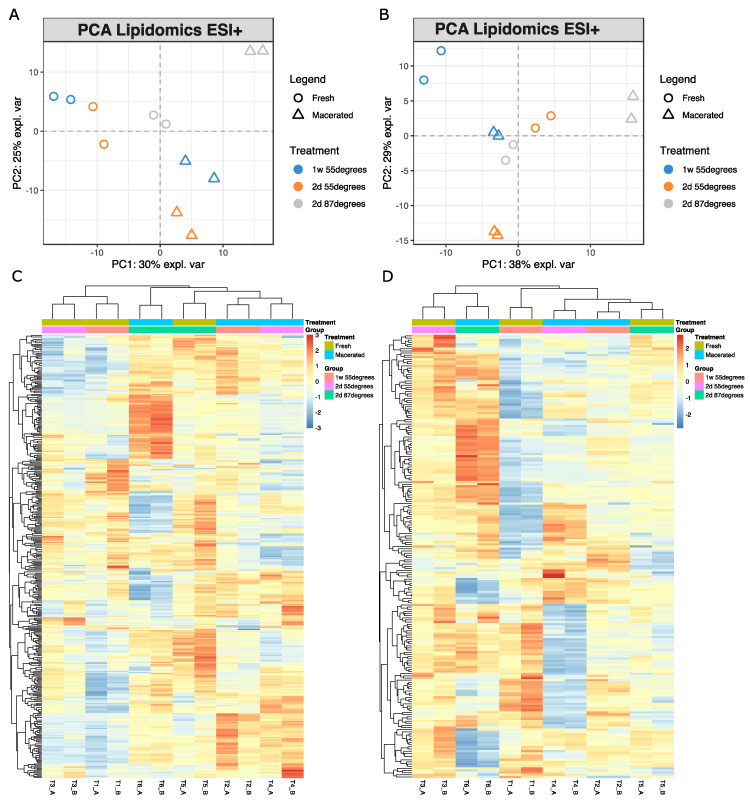
PCA plots lipid profiling in ESI+ (**A**) and ESI− (**B**) show a good separation between each treatment, while the separation between fresh and macerated specimens is less clear than in the metabolomics profile. Hierarchical clustering and heatmaps (**C**,**D**) show groups of macerated and non-macerated samples not clustering together.

Moving to the lipidomic profiling, for both ESI+ (Figure 4A) and ESI− (Figure 4B), the two groups (fresh vs. macerated) are less clustered than in the metabolomics assays, despite the cumulative variance explained is, respectively, 55% and 67%. In ESI+, there is a separation on the first PCA component especially between the samples macerated with the protocol #3 and the fresh ones, whereas the other macerated ones are located closer to the fresh ones on the first component, despite their separation on the second component. Additionally, in this case, consistently with what observed for the metabolomic results, the bone processed with protocol #3 shows a much clearer separation from the remaining bones treated with protocols #1 and #2 and from the fresh ones, with a clear distinctive pattern also in the respective heatmap (Figure 4C). In ESI−, PCA shows that bones treated with protocol #1 cluster fresh bones, whereas bones macerated with the other protocols cluster in other quadrants, overall complicating the interpretation of the obtained results. When considering the hierarchical clustering (Figure 4D), two fresh bones and the two specimens processed with protocols #1 and #2 form a cluster, whereas the bone treated with protocol #3 are in a separate cluster together with one of the three untreated bones. According to the heatmap, protocols #1 and #2 share similar profiles, that appear to be distinct from the fresh bones despite their positioning in the tree. These results suggest that differences in the lipidic content are not related exclusively to the maceration protocols tested, and that additional variables may play a role in the post-maceration lipidomic signature. No lipid compounds are seen to behave consistently according to the three different maceration protocols, despite it is noticeable a class of lipids more abundant only in the samples processed with protocol #3 in both ionization modes (Figure 3C,D). Considering only the differences between fresh and macerated bones in ESI+, also due to the larger sample size, *t*-test reveals that acetyl-CoA (AcCa), ceramides (Cer), phosphatidylethanolamines (PE), and phosphatidylserines (PS) are the classes of lipids significantly more abundant in the fresh samples (Table S1. In the macerated sample there is higher abundance of several triglycerides (TG) of variable chain length, sphingomyelins (SM), phosphatidylinositols (PI), phosphatidylcholines (PC), two diglycerides (DG) and one compound for lysophosphatidylcholine (LPC), lysophosphatidylethanolamine (LPE), and lysophosphatidylserine (LPS). Boxplots and *t*-test results are reported, respectively, in Figure S2 and Table S1. Regarding lipidomics ESI− (Figure S3 and Table S1), significant differences were seen for ceramides with higher abundances for the fresh samples. The same trend was seen for PSs and one LPS. In contrast, LPCs, LPEs, PCs and PIs show higher abundances in the macerated samples confirming what also shown by lipids profiled in ESI+ mode.

## 4. Discussion

This pilot study demonstrates how procedures normally adopted by some HFTFs to clean and prepare human bones for long-term storage may impact the ability to perform biomolecular analyses aimed at obtaining reliable and unbiased results from bones for PMI estimation. The increased interest in metabolomics and lipidomics applied to the forensic field, in addition to the technological developments in mass spectrometry that have characterized the recent years, have started to pose questions on the reliability of the biomolecular results obtainable from the skeletal collections at the HFTFs. Due to the small sample size, results of the present study are considered to be preliminary, and the overall conclusions of the study are uniquely intended to be a proof-of-concept to start building awareness around this topic, which has never been considered a major issue until now. Therefore, statistical analyses were used more as a guide in the interpretation of the results rather than a tool to draw conclusive evidence, and further studies on a larger sample size, ideally human, will be beneficial to expand on these findings. We used an animal model to test three different maceration protocols used at HFTFs and then applied a high-sensitivity LC-MS/MS approach for small compounds and lipids profiling. As expected, our results show a general decrease in the normalized intensities of all the endogenous metabolites (e.g., amino acids and nucleobases) as well as the presence of new compound associated with the chemical detergents used during the cleaning process (e.g., surfactants). Changes in the lipidomic profile resulted in being the most complex to interpret, with certain classes being more abundant in the macerated samples and with others being more intense in the fresh ones. Despite the use of animal proxies has been largely employed in forensic sciences, there are several factors that could not be controlled and could have an impact on the outcome of the study. Amongst those there are nutrition, health status and age of the animal. However, due to the source of the bones employed in the experiment (butchered animals to be used for human consumption) it is reasonable to assume that all animals were similarly fed, treated, shared good health conditions and were slaughtered at the same age. This is supported by the data shown in Figure 2 and Figure 4, where the fresh individuals closely clustered together, suggesting overall a minimal interindividual variability.

When considering the metabolites, the PCA score plot (Figure 2A) revealed that specimens treated using protocol #1 and #2 differ significantly from protocol #3. It is reasonable to think that the high temperature employed (87 °C compared to 55 °C), combined with the different type of detergent used, drives the variations observed, and that the length of the procedure (two days vs. one week) affects the results only marginally. There is evidence in literature supporting the fact that high temperatures may induce significant changes in the chromatographic pattern of the Metabolome. Fang et al. [19] tested human serum by means of LC-MS/MS after exposing the material to temperatures ranging between 60 °C and 250 °C for an exposure time up to 300 s and proved that the harsher treatment induced alterations in up to 40% of the chromatographic peaks. Nucleosides and nucleotides were transformed into pure derivates, while tri- and di-organophosphates into their mono-components [19]. Moreover, it must be noted that water, in general and regardless of its temperature, poses issues for metabolomic analyses when samples are submerged prior to analysis. In fact, water is the main polar solvent used for metabolite extraction, including the Folch extraction protocol [20] employed in the present study, and it is not surprising therefore that submerging bones in water as a pre-treatment, can severely affect their metabolomic profiles. The potential release of small molecules and compounds in solution during the maceration of the bones can explain why, in general, most of the endogenous metabolites are observed only in the non-macerated samples and not in their treated counterparts. In addition to the intrinsic metabolites, cannabidiol, 1-tetradecylamine bis(2-ethylhexyl) amine, N,N-dimethyldecylamine, N-oxide, dodecyltrimethylammonium, xylenesulfonate, (16R,17S)-8-(4-morpholinyl), dodecyl sulfate and myristyl sulfate were observed specifically in the samples treated with protocols #1 and #2, that share the same detergent, showing that traces of the detergent used during the maceration process can be identified in the cortical portion of the bones, altering the original metabolomic fingerprint of the specimen. Similarly, for protocol #3 (where a different cleaning agent was used), other exogenous contaminants such as 4-Guanidinobutyric acid, (+/−)13-HODE, (+/−)9,10-dihydroxy-12Z-octadecenoic acid, neopterin, and CAPSO were identified. *t*-tests run on the single metabolites when comparing fresh and macerated samples further support the hypothesis that maceration can induce not only the loss of specific metabolites, but also introduce specific contaminants depending on the chemicals used. The investigation of the changes associated with the metabolome is popular in food science and can be related to this work. For example, an experiment run to estimate the ideal combination of cooking temperature and duration for Thai chicken bone soup showed an increase in the abundance of free amino acid (AAs) and essential AAs (EAAs) in the bone broth following boiling, with an associated and expected decrease in their concentration in the bone tissue [22]. This fully supports our findings, confirming a general decrease in the endogenous metabolites following maceration, as shown in Figure 3.

Previous works conducted on several soft tissues in forensic science highlighted the great potential for metabolomics to reveal information on PMI [7,23,24]. Despite bones may offer a similar success rate when analyzed via metabolomics for PMI, the changes introduced by these protocols can significantly affect their metabolomic signature, consequently posing severe limitations on the use of treated osteological collection for this purpose. As an example, hypoxanthine, choline, creatine, and betaine have been previously found to be good candidates for PMI estimation in vitreous humor [24,25]. Similarly, xanthine, uracil, choline phosphate, and 1-methylnicotinamide have been identified as potential bio markers for PMI estimation in muscle tissue [7]. Our study showed that these metabolites are also present in the non-treated bone (Figure 3), and that their abundance is severely affected by the maceration treatment. Therefore, these molecules may lose their predictive power for PMI when bones are treated prior long-term storage. As a result, it is of primary importance to ensure the best possible post-mortem preservation of metabolites in bones, reducing the invasiveness of any procedure related to bone tissue handling and cleaning to allow an optimal performance of the subsequent biochemical studies.

Despite single lipid compounds do not seem to change consistently according to the different protocols used, certain lipid classes show a more regular pattern when considering either fresh or macerated samples. The separation between pre- and post-treatment samples, as well as those existing between different protocols, are better appreciated in ESI+ mode. In fact, when investigated in ESI−, the modifications induced by the maceration processes do not show clear distinctions between any of the groups, including fresh vs. macerated. In a previous study, Nawar et al. [18] showed that lipids in glyceride mixtures are unaltered at 60 °C, showing that the thermal hydrolysis of triglycerides (TG) results in the preferential release of shorter-chains TG and of unsaturated fatty acids [18]. More recently Charuwat et al. [17] tested the effect thermal hydrolysis between 90–150 °C and 160–250 °C for a maximum of eight hours on long chain fatty acids in sludges by means of gas chromatography. Results showed that long chain fatty acids are resistant to thermal hydrolysis treatments, with approximately only 1% of saturated fatty acids becoming shortened at 160 °C [17].

It is difficult to determine if a shortening of the lipid chains was directly linked with the maceration processes due to the experimental set up. However, the observed modification in the lipidome could be potentially linked to the fact that the bones used in the experiment were fresh and had some remaining adhering soft tissues and blood on their surface. When macerating them at temperatures above 50 °C, some lipid classes originated from these remaining soft tissues may have been released in the water bath and may have eventually adhered onto the periosteal surface, increasing therefore the abundance of these compounds specifically in the macerated bones, particularly at higher temperatures (87 °C, Figure 4). Table 2 summarizes the most striking outcome of the study highlighting those certain classes of lipids specifically increase or decrease after the treatment. Generally, AcCA, Cer, PE, and PS are less abundant in the macerated samples, while the opposite trend is true for the remaining classes in both ionization modes.

Despite the limited body of research in the application of lipidomics analyses to forensics, several studies have shown the potential of lipidomics for PMI estimation from several tissues. Wood and Shirley [8] sampled the anterior quadriceps muscle from one donated cadaver 1, 9, and 24 days post-mortem, and showed the decline of sterol sulphates, choline plasmalogens, ethanolamine plasmalogens, and phosphatidylglycerols and the contemporaneous increase in free fatty acids with increasing PMIs, indicating that the decrease in membrane glycerophospholipids was the best indicator for PMI estimation. To our knowledge, only one other study has conducted analyses on lipids extracted from bone material for forensic applications. Dudzik et al. [26] analyzed dry trabecular bone marrow cores in their native state at the time of death and specific timepoints for up to two years post-mortem from vertebral bodies, tibiae, and calcanei of human donated cadavers. With direct infusion MS they identified stable lipids belonging to 56 lipid subclasses. after approximately seven years retrieving 76 potential N-acyl amino acids that could serve for PMI estimation. The same authors following-up the previous work [10] analyzed the trabecular bone of calcaneus, proximal tibia, and 4th lumbar vertebra from 20 fresh donors with a maximum PMI of 24 months collecting samples every six months. Additionally, they analyzed a cross-sectional sample by coring bones belonging to 130 individuals curated in the William Bass Skeletal collection, with PMIs ranging from 3–37 years. Results from the calcanei showed an increase in PC 34:1 and decrease in PC 36:1 and PC 36:2 up to 12 months PMI. Once again, PC is one of the lipid classes affected by all the maceration procedures employed in the present study. More generally, they showed that the first three months are crucial for the degradation of phosphatidylcholines in bones, after which PCs reach a plateau, making them less effective for prolonged PMI estimations [10]. Despite the non-treated bones in the study (e.g., those sampled from the decomposing cadavers) may have given reliable observations, it is difficult to exclude that the relatively stable abundances of PCs observed by Dudzik et el. [10] in the samples originated from the curated collection were not linked to the maceration treatments used at the facility, as specifically observed in this study for PCs. Therefore, additional studies on non-treated remains for prolonged PMIs (e.g., >2 years) may be crucial to shine a light on this topic and to clarify which compounds may be the best indicators for PMI estimation in bones. Despite metabolomics is not yet at a stage in which could be applied to forensic investigations, the development of new methods profoundly depends on the analysis of taphonomically “controlled” human material, that can therefore only be sourced from HFTFs. This undoubtedly demonstrates the potential that lies in the skeletal collections provided by these facilities, and the consequent necessity to standardize the bone cleaning procedures aiming for the creation of long-term skeletal collections of which biomolecular and biochemical profiles are not, or are minimally (and in a controlled way), affected by the treatment protocols.

## 5. Conclusions

The present study provides preliminary results on the changes in both metabolite and lipid profiles induced by some of the maceration procedures commonly used in HFTFs to re-move the superficial soft tissues prior to long term bone storage. The results presented here on the metabolomic and lipidomic alteration of the profiles of chemically and thermally treated bones in comparison with their non-treated counterparts highlight the importance of minimizing the loss of the biomolecular integrity when using soft tissue removal protocols for skeletal preparation. We have shown that these treatments induce not only the loss of endogenous metabolites, but also introduce contaminations caused by the chemicals used during these processes. The high-water temperature and the type of chemical used in the water bath are the main variables able to affect the bone metabolome of treated bones. However, also less harsh protocols involving lower water temperatures and shorted durations can affect irreversibly the bone metabolome. The bone lipidome seems to be less affected by the submersion in lower temperatures and more impacted when higher temperatures are used. Furthermore, it may be susceptible to potential contaminations introduced by the presence of residual soft tissues and blood in the water bath. The employment of various protocols in different institutions drastically reduces the possibility to compare results from experiments on material originated from different HFTFs, with clear negative implications for the forensic community. Therefore, a more standardized way of processing bones should be sought after to preserve the invaluable potential that human donated cadavers offer to science. However, treated bones may not be suitable for any metabolomic nor lipidomic analysis aimed at addressing PMI for forensic applications, and further analyses on human bones may validate these findings. Alternatives, such as performing small bone samplings prior to the maceration of the remains to allow future molecular analyses on those samples, should be considered by HFTFs to maximize the amount of information recoverable from bones when maceration cannot be avoided. Nonetheless, cooperation between the HFTFs is a central necessity in order to let the field of forensic science grow and, with it, the breadth of studies based on their skeletal collections.

## Figures and Tables

**Table 1:** Maceration protocols employed in the study.

Bone	Protocol	Temperature	Duration	Cleaning Agent	Source
T1A,B	-	-	-	-	-
T2A,B	#1	55 °C	1 week	Dish soap:deionized water	STAFS
T3A,B	-	-	-	-	-
T4A,B	#2	55 °C	2 days	Dish soap:deionized water	STAFS
T5A,B	-	-	-	-	-
T6A,B	#3	87 °C	2 days	Laundry detergent:deionized water	FACTS

**Table 2:** Variation trends for lipid classes induced by the maceration process based on *t*-test results between fresh and macerated samples without considering different protocols (↓ = decreasing trend; ↑ = increasing trend).

ESI	Lipid Class	Variation Trend with Treatment
Positive	Acetyl-CoA (AcCa)	↓
	Ceramide (Cer)	↓
	Phosphatidylethanolamine (PE)	↓
	Phosphatidylserine (PS)	↓
	Sphingomyelin (SM)	↑
	Phosphatidylinositol (PI)	↑
	Triglyceride (TG)	↑
	Phosphatidylcholine (PC)	↑
	Diglyceride (DG)	↑
	Lysophosphatidylcholine (LPC)	↑
	Lysophosphatidylethanolamine (LPE)	↑
Negative	Ceramides (Cer)	↓
	Phosphatidylserines (PS)	↓
	Lysophosphatidylserine (LPS)	↓
	Lysophosphatidylcholine (LPC)	↑
	Phosphatidylcholines (PC)	↑
	Phosphatidylinositols (PI)	↑

## Data Availability

The raw data used in this manuscript available via the EMBL-EBI’s MetaboLights repository [27] under the permanent unique identifier MTBLS5561 https://www.ebi.ac.uk/metabolights/MTBLS5561 accessed on 1 October 2022.

## Supplementary Materials

The following supporting information can be downloaded at: https://www.mdpi.com/article/10.3390/metabo12111020/s1, Figure S1: Boxplots for metabolomics ESI−; Figure S2: Boxplots for lipidomics. ESI+; Figure S3: Boxplots for lipidomics ESI−; Table S1: *t*-test results for differences between fresh vs. macerated samples.
