# Supplementary material for: Preliminary Investigation of the Effect of Maceration Procedures on Bone Metabolome and Lipidome
Source: Metabolites. 2022 Oct 25;12(11):1020. doi: 10.3390/metabo12111020 (PMC9693520; doi:10.3390/metabo12111020)
Supplement: Supplementary file 1 [file metabolites-12-01020-s001.zip › metabolites-1981074-Supplementary-Figures.pdf]

Preliminary Investigation of the Effect of Maceration Procedures on Bone Metabolome and Lipidome

Andrea Bonicelli [1,†], William Cheung [2], Sheree Hughes [3], Daniel J. Wescott [4] and Noemi Procopio [1,4,*,†]

[1] The Forensic Science Unit, Faculty of Health and Life Sciences, Ellison Building, Northumbria University, Newcastle Upon Tyne NE1 8ST, UK
[2] Department of Applied Sciences, Faculty of Health and Life Sciences, Northumbria University, Newcastle Upon Tyne NE1 8ST, UK
[3] Department of Forensic Science, College of Criminal Justice, Sam Houston State University, Huntsville, TX 773402525, USA
[4] Forensic Anthropology Center at Texas State, Department of Anthropology, Texas State University, San Marcos, TX 78666, USA
* Correspondence: nprocopio@uclan.ac.uk
† Current Affiliation: School of Natural Sciences, University of Central Lancashire, Preston PR1 2HE, UK.

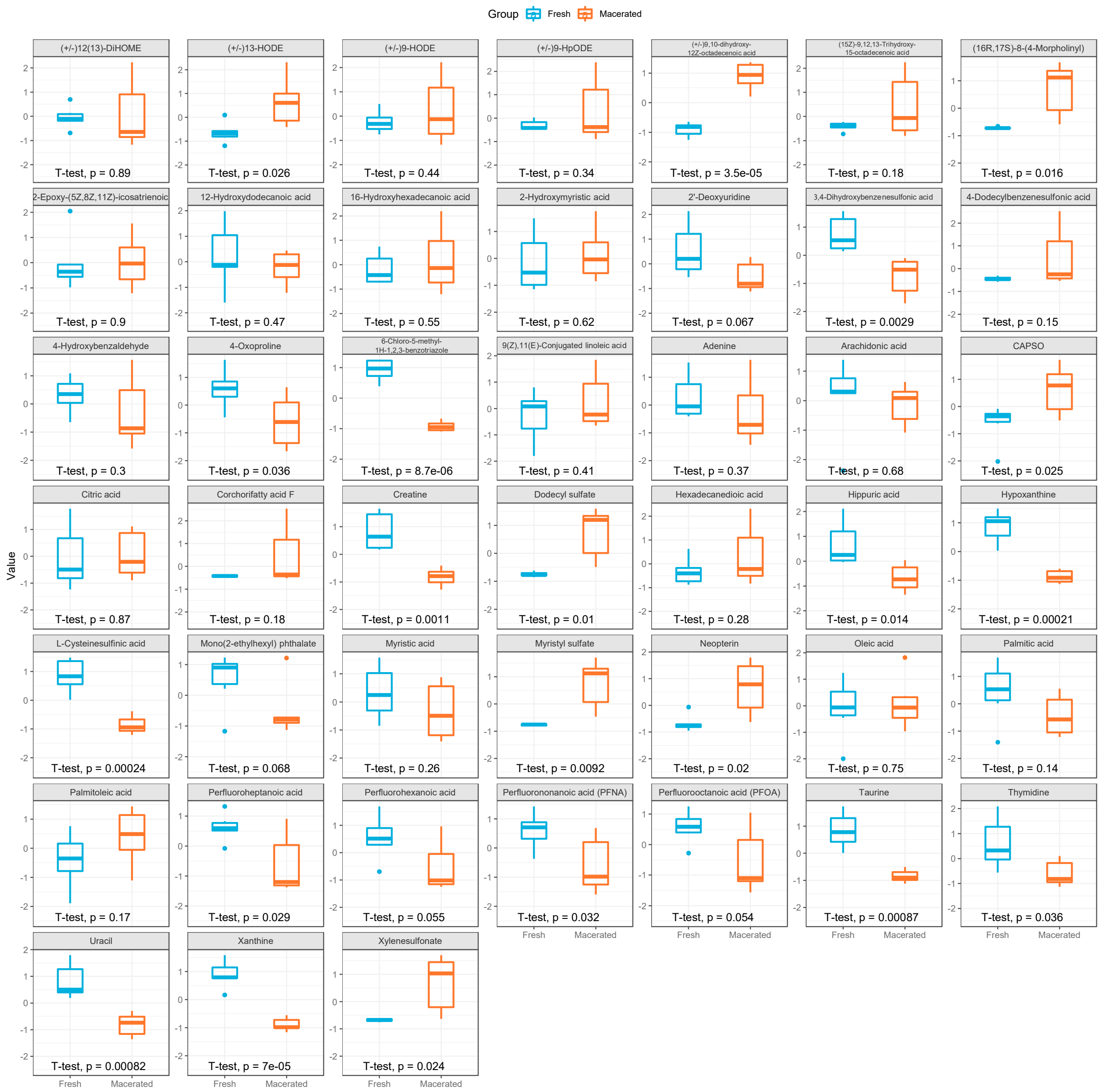

Figure S1. Boxplot showing metabolites profiled in ESI- and p-value for t-test.

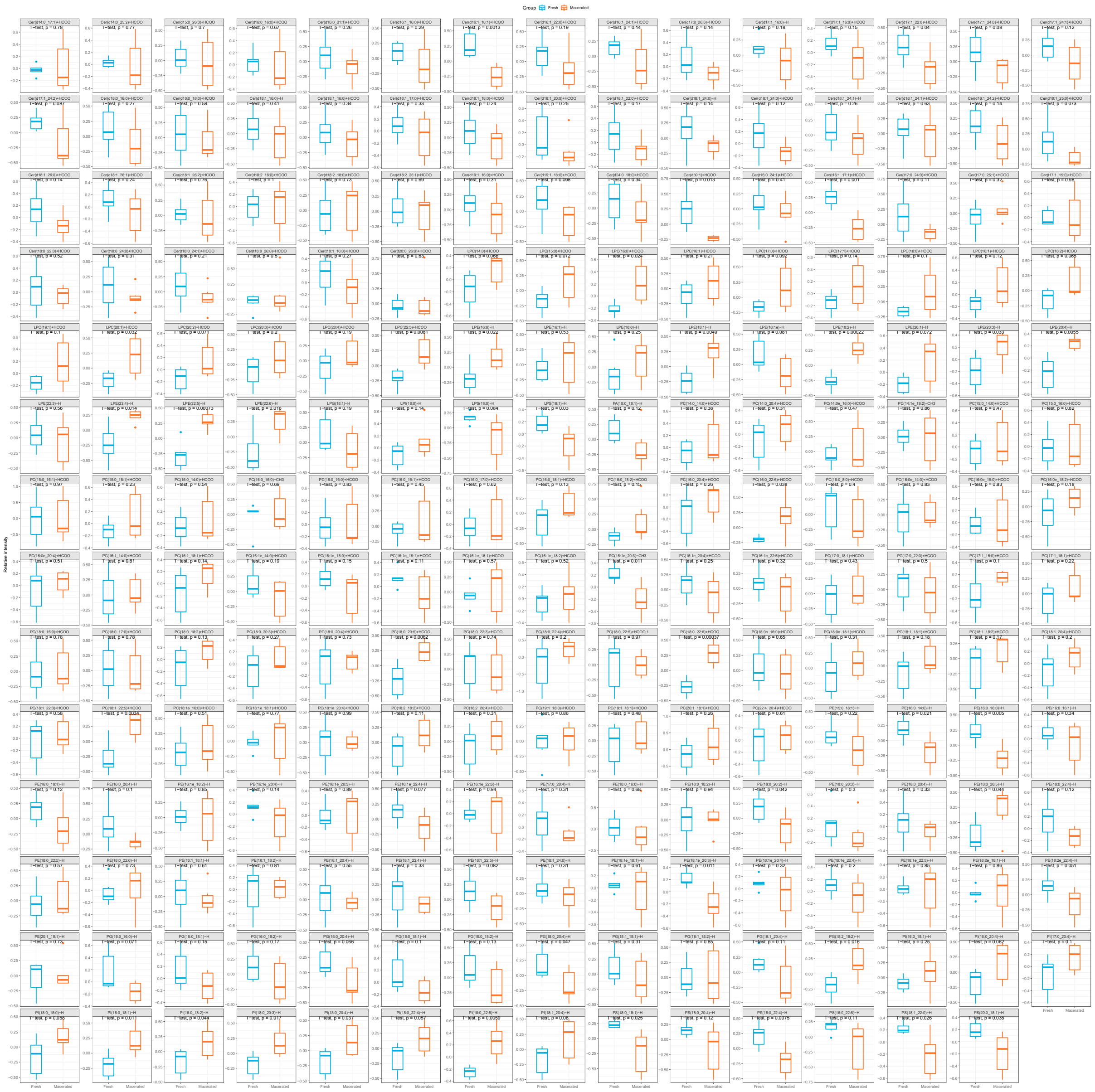

Figure S2. Boxplot showing Lipids profiled in ESI+ and p-value for t-test.

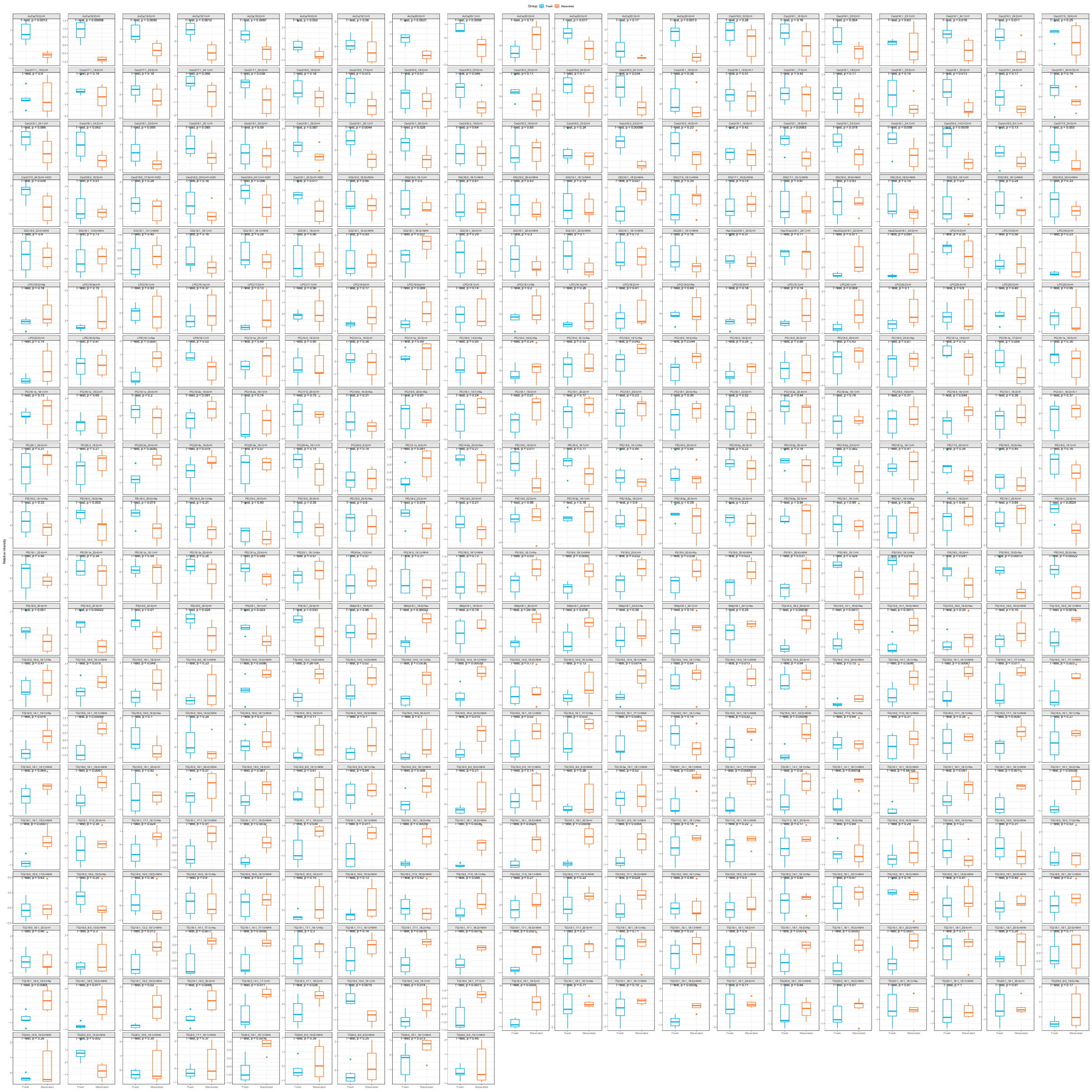

Figure S3. Boxplot showing Lipids profiled in ESI- and p-value for t-test.
